# Bleeding events associated with a low dose (110 mg) versus a high dose (150 mg) of dabigatran in patients treated for atrial fibrillation: a systematic review and meta-analysis

**DOI:** 10.1186/s12872-017-0511-8

**Published:** 2017-03-15

**Authors:** Pravesh Kumar Bundhun, Nabin Chaudhary, Jun Yuan

**Affiliations:** 1 Institute of Cardiovascular Diseases, The First Affiliated Hospital of Guangxi Medical University, Nanning, Guangxi 530021 People’s Republic of China; 2 Department of Cardiology, The People’s Hospital of Guangxi Zhuang Autonomous Region, Nanning, Guangxi 530021 China

**Keywords:** Atrial fibrillation, Dabigatran, Bleeding events, Stroke, Minor bleeding

## Abstract

**Background:**

The newer oral anticoagulant dabigatran is considered to be more beneficial in patients with non-valvular Atrial Fibrillation (AF) when compared to warfarin. However, because bleeding events which are associated with a low dose (110 mg) versus a high dose (150 mg) of dabigatran have seldom been compared, we aimed to systematically solve this important issue through this meta-analysis.

**Methods:**

English publications comparing 110 mg with 150 mg dabigatran in patients who were treated for AF were electronically searched through medical databases. Bleeding outcomes were the major clinical endpoints to be assessed. Odds Ratios (OR) and 95% Confidence Intervals (CIs) for each subgroup were calculated and the main analysis was carried out by the latest version of the RevMan 5.3 software.

**Results:**

Twenty-nine thousand two hundred and sixty-four (29,264) patients were included in this meta-analysis. Fifteen thousand eight hundred and forty-eight (15,848) patients were treated with 110 mg dabigatran whereas 13,416 patients were treated with 150 mg dabigatran. 110 mg dabigatran was associated with a significantly lower rate of minor bleeding (OR: 1.19, 95% CI: 1.10–1.27; *P* < 0.00001). A similar rate of fatal and major bleeding was observed with both dosages (OR: 1.12, 95% CI: 0.69–1.82; *P* = 0.65) and (OR: 1.09, 95% CI: 0.86–1.37; *P* = 0.49) respectively. However, ischemic stroke insignificantly favored a higher dose of dabigatran, (OR: 0.77, 95% CI: 0.51–1.16; *P* = 0.21). In addition, this analysis showed mortality to significantly favor 150 mg of dabigatran (OR: 0.41, 95% CI: 0.34–0.50; *P* < 0.00001).

**Conclusion:**

No significant differences in major and fatal bleedings were observed with 110 mg versus 150 mg dabigatran. However, 110 mg dabigatran was associated with a significantly lower risk of minor bleeding. These results should further be confirmed in future trials.

## Background

Stroke is very common in patients with non-valvular atrial fibrillation (AF) [1]. Therefore, as a secondary prevention of this critical health condition, lifelong warfarin has often been recommended in several hospitals [2]. However, because warfarin also has limitations, and since it is contraindicated in certain subgroups of patients, the development of newer oral anticoagulants was urgently required [3]. In the United States, according to current practice guidelines, dabigatran is nowadays recommended for stroke prevention in patients with similar conditions. Dabigatran 110 mg (low dosage) or dabigatran 150 mg (high dosage) twice daily is often required [4]. Several studies showed dabigatran to be more beneficial when compared to warfarin [5–7]. However, since these studies have seldom compared bleeding events which are associated with a low dose (110 mg) versus a high dose (150 mg) of dabigatran in patients who were treated for non-valvular AF, we aimed to systematically solve this important issue through this meta-analysis.

## Methods

### Searched databases and searched terms

Electronic databases including Medline (National Library of Medicine), EMBASE and the Cochrane library of Randomized Controlled Trials were searched for relevant English language publications comparing 110 mg with 150 mg dabigatran in patients who were treated for AF. The terms ‘dabigatran and bleeding’ and ‘dabigatran and atrial fibrillation’ were used during this search process. Because dabigatran is a new oral anticoagulant when compared to warfarin, the words ‘new oral anti-coagulant’ and the abbreviations ‘NOAC’ as well as ‘AF’ were also used. To further enhance this search process, relevant terms such as ‘non-valvular atrial fibrillation’, ‘dabigatran and stroke’, ‘dabigatran and outcomes’ were also used.

### Inclusion and exclusion criteria

Studies were included if:

- They were randomized controlled trials (RCTs) or observational studies comparing patients who were treated with 110 mg versus 150 mg dabigatran.
- They involved patients with non-valvular AF.
- They reported at least bleeding outcomes (which could be any bleeding event whether major bleeding, minor bleeding, fatal bleeding, and so on) as their clinical endpoints.

Studies were excluded if:

- They were meta-analyses or case studies even if their main focus was on dabigatran.
- They compared warfarin versus dabigatran, but however, the two different dosages of dabigatran were not compared (110 mg dabigatran was not compared with 150 mg dabigatran).
- They did not report bleeding events (any type) among their clinical endpoints.
- They were duplicates of the same publication which were obtained from different databases or they were different studies which involved the same trial.

### Definitions and outcomes

The outcomes which were analyzed have been summarized in Table 1.

**Table 1:** Reported outcomes

Studies	Reported outcomes	Follow up periods
Connolly 2013 [22]	Stroke or systemic embolism, ischemic stroke, hemorrhagic stroke, MI, pulmonary embolism, major bleeding, life-threatening bleeding, GI bleeding, intracranial bleeding, extracranial bleeding, fatal bleeding, minor bleeding, total mortality	2.3 years
Eikelboon 2013 [23]	Major bleeding, intracranial bleeding, intracerebral bleeding, extracranial bleeding, GI bleeding, life-threatening bleeding, fatal bleeding, minor bleeding	2 years
Larsen 2013 [24]	Stroke, systemic embolism, intracranial bleeding, total mortality, GI bleeding, major bleeding, MI, pulmonary embolism	≥1 year
Maura 2015 [25]	Bleeding events, ischemic stroke or systemic embolism	3 months
Nishtala 2016 [26]	Intracerebral bleeding, GI bleeding	30 days

Abbreviations: *GI* gastro-intestinal, *MI* myocardial infarction

Bleeding events (including major bleeding, minor bleeding, GI bleeding, intracerebral bleeding, extracranial bleeding, fatal bleeding and life-threatening bleeding) were considered as the primary outcomes in this analysis, whereas any other adverse clinical outcomes such as mortality, myocardial infarction (MI), pulmonary embolism, stroke and systolic embolism were considered as the secondary outcomes.

According to Table 1, major and minor bleeding were each reported in three studies respectively. Fatal and life-threatening bleeding were each reported in two studies respectively whereas GI bleeding was reported in four studies. Moreover, intracranial bleeding and extracranial bleeding were reported in three and two studies respectively.

Mortality, pulmonary embolism and MI were reported each in two studies respectively. Stroke or systemic embolism was reported in three studies.

#### Major bleeding

Clinically overt bleeding which was associated with any of the following: fatal or severe outcome, involvement of a main anatomic site, decrease in hemoglobin concentration of more than 2 g/dL with reference to the actual value, transfusion of more than 2 units of whole blood or packed red blood cells, or any permanent disability.

#### Minor bleeding

Other overt bleeding circumstances that did not meet the criteria for major or clinically relevant non-major bleeding as stated.

#### Life threatening bleeding

Involved fatal bleeding, symptomatic intracranial bleeding, bleeding with a decrease in the hemoglobin level ≥50 g/L, or bleeding that required transfusion of ≥4 U of blood or bleeding that necessitated surgery.

### Data extraction

Eligible studies were independently reviewed and assessed by three authors (PKB, NC and JY). Data regarding the total number of patients who were treated with 110 mg and 150 mg dabigatran respectively, the baseline characteristics of the patients and the adverse clinical events reported with their respective duration of follow up periods, were systematically extracted. Disagreements which followed were discussed and resolved by an agreement among the authors. The PRISMA guideline was followed [8, 9].

### Statistical analysis and interpretations

Subgroup analysis was expected to show heterogeneous results. Therefore, heterogeneity was interpreted using the Cochrane Q-statistic and the I^2^-statistic tests respectively.

In this analysis, a *P* value referring to ≤0 · 05 was considered statistically significant whereas any outcome analysis with a *P* value larger than 0.05 was not considered significantly different.

I^2^ was interpreted in the following ways: an I^2^ with a low value indicated a low heterogeneity whereas an I^2^ with an increasing percentage indicated highly heterogeneous results.

Fixed effects model (I^2^ < 50%) and Random effects model (I^2^ > 50%) was dependent on the I^2^ value obtained.

In this analysis, only a few studies which satisfied the inclusion and exclusion criteria were considered relevant and therefore, publication bias could easily be assessed by visually observing funnel plots generated by the RevMan software.

The latest version of RevMan (5.3) was used to generate Odds Ratios (OR) and 95% Confidence Intervals (CIs).

Ethical approval was not required for this type of study.

## Results

### Result of the searched strategy

**Fig. 1:**
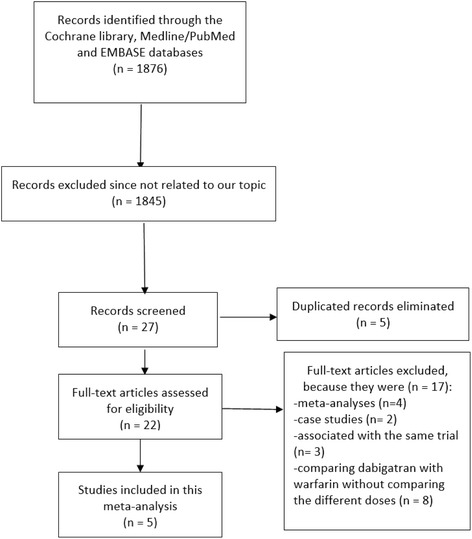
Flow chart representing the study selection

One thousand eight hundred and seventy-two (1872) articles were obtained through the previously-mentioned electronic databases. One thousand eight hundred and forty-five (1845) articles were eliminated since they were not directly related to the scope of this research. Among the 27 remaining studies, five (5) publications were eliminated since they were duplicates of the same study which were repeatedly obtained from different searched databases. Moreover, four (4) other articles which were meta-analyses comparing warfarin with other different oral anticoagulants including dabigatran and two (2) articles which were case studies were also eliminated. A further eleven (11) articles were eliminated since three (3) among them involved the same trial whereas eight (8) studies compared warfarin with dabigatran without further comparing high dose with low dose dabigatran. Finally, five (5) studies were included in this meta-analysis (Fig. 1).

### General features of the studies which were included in this analysis

The types of study reported, the patients’ enrollment periods, and the total number of patients treated with 110 and 150 mg dabigatran have all been summarized in Table 2.

**Table 2:** General features of the studies which were included in this meta-analysis

Studies	Type of study	Patients’ enrollment	No of patients treated with 110 mg dabigatran (n)	No of patients treated with 150 mg dabigatran (n)	NVAF
Connolly2013 [22]	RCT	2008–2012	2914	2937	+
Eikelboon2013 [23]	RCT	2005–2009	6015	6076	+
Larsen2013 [24]	Observational	2011–2012	2326	1760	+
Maura2015 [25]	Observational	2011–2012	1198	490	+
Nishtala2016 [26]	Observational	2011–2012	3395	2153	+
Total no of patients (n)			15,848	13,416	

Abbreviations: *RCT* randomized controlled trials, *AF* atrial fibrillation, *NVAF* non-valvular atrial fibrillation

A total number of 29,264 patients were included in this analysis. Fifteen thousand eight hundred and forty-eight (15,848) patients and 13,416 patients were treated with 110 mg versus 150 mg dabigatran respectively. As shown in Table 2, two studies were randomized trials whereas the remaining three studies were observational studies. Patients’ enrollment period ranged from the year 2005 to 2012.

### Baseline characteristics

Table 3 shows the baseline features of the studies which were included in this meta-analysis.

**Table 3:** Baseline characteristics of the studies which were included in this analysis

Studies	Mean age (y)	Males (%)	Hypertension (%)	DM (%)	Previous stroke/TIA (%)
	110/150 mg	110/150 mg	110/150 mg	110/150 mg	110/150 mg
Connolly2013 [22]	71.0/71.0	66.0/65.0	80.0/78.0	23.0/22.0	20.0/21.0
Eikelboon2013 [23]	71.4/71.5	64.3/63.2	78.8/78.9	23.4/23.1	19.9/20.3
Larsen2013 [24]	74.7/67.4	46.9/61.5	18.0/22.7	10.8/12.1	17.5/17.1
Maura2015 [25]	77.4/66.1	48.0/66.0	83.0/74.0	20.0/18.0	8.0/6.0
Nishtala2016 [26]	77.3/77.3	53.1/53.1	-	15.6/15.6	18.8/18.8

Abbreviations: *y* year, *DM* diabetes mellitus, *TIA* transient ischemic attack, *110 mg* 110 mg of dabigatran, *150 mg* 150 mg of dabigatran

According to Table 3, the patients had a mean age above 65 years old. Majority of the patients were males. Study larsen2013 consisted of a lower percentage of patients with hypertension. In addition, the percentage of patients with diabetes mellitus varied from 10.8 to 23.1%. Almost 20% of patients were previously affected by stroke in both groups (110 mg dabigatran and 150 mg dabigatran). Overall, there were no significant differences observed in baseline features among patients who were treated with 110 mg dabigatran and 150 mg dabigatran.

### Bleeding events which were associated with a low versus a high dose of dabigatran

**Fig. 2:**
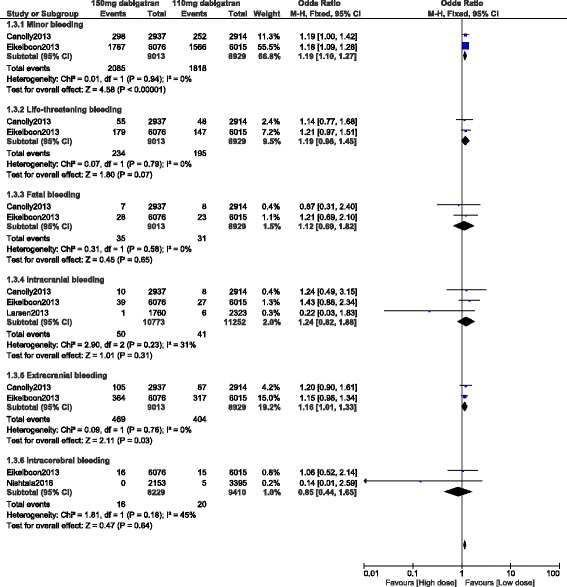
Bleeding events associated with 110 mg versus 150 mg dabigatran (part 1)

Results of this analysis showed that 110 mg dabigatran was associated with a significantly lower rate of minor bleeding (OR: 1.19, 95% CI: 1.10–1.27; *P* < 0.00001). Life-threatening bleeding insignificantly favored a low dose of dabigatran (OR: 1.19, 95% CI: 0.98–1.45; *P* = 0.07). Moreover, a similar rate of fatal and major bleeding was observed whether with a low dose (110 mg) or a high dose (150 mg) of dabigatran (OR: 1.12, 95% CI: 0.69–1.82; *P* = 0.65) and (OR: 1.09, 95% CI: 0.86–1.37; *P* = 0.49) respectively. Extracranial bleeding significantly favored a lower dose of dabigatran (OR: 1.16, 95% CI: 1.01–1.33; *P* = 0.03). However, intracranial and intracerebral bleeding were not significantly different in these patients who were treated with these two different dosages of dabigatran (OR: 1.24, 95% CI: 0.82–1.88; *P* = 0.31) and (OR: 0.85, 95% CI: 0.44–1.65; *P* = 0.64) respectively. These results have been illustrated in Fig. 2.

**Fig. 3:**
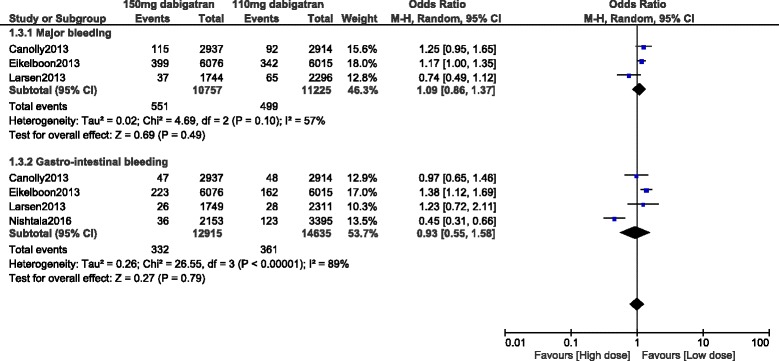
Bleeding events associated with 110 mg versus 150 mg dabigatran (part 2)

Major bleeding and GI bleeding were also similarly observed with 110 mg and 150 mg dabigatran (OR: 1.09, 95% CI: 0.86–1.37; *P* = 0.49) and (OR: 0.93, 95% CI: 0.55–1.58; *P* = 0.79) respectively. However, an increased heterogeneity was observed while analyzing these two subtypes of bleeding. These results have been represented in Fig. 3.

**Fig. 4:**
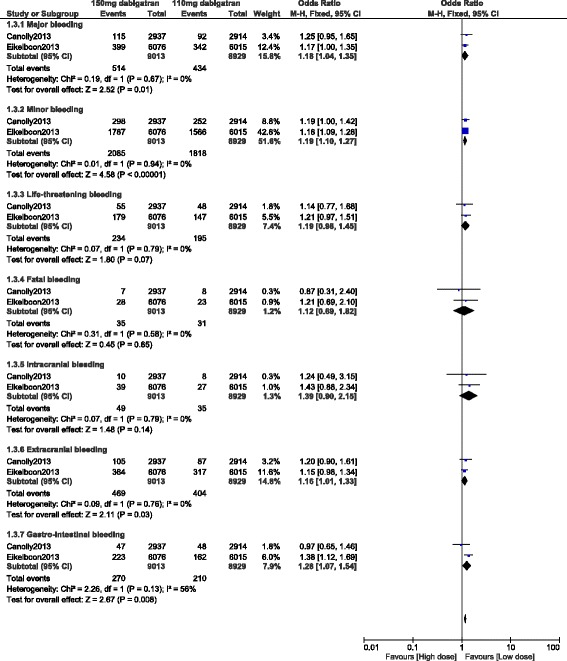
Bleeding events associated with 100 mg versus 150 mg dabigatran using data obtained only from randomized trials

Since observational studies might lead to the introduction of bias, another analysis was carried out only using data which were obtained from randomized trials. Results of this specific analysis showed 110 mg dabigatran to be associated with significantly lower major bleeding (OR: 1.18, 95% CI: 1.04–1.35; *P* = 0.01). Minor bleeding, extracranial bleeding and gastrointestinal bleeding also significantly favored a lower dose of dabigatran (OR: 1.19, 95% CI: 1.10–1.27; *P* < 0.00001), (OR: 1.16, 95% CI: 1.01–1.33; *P* = 0.03) and (OR: 1.28, 95% CI: 1.07–1.54; *P* = 0.008) respectively. However, the results for life-threatening bleeding, fatal bleeding and intracranial bleeding were not statistically significant (OR: 1.19, 95% CI: 0.98–1.45; *P* = 0.07), (OR: 1.12, 95% CI: 0.69–1.82; *P* = 0.65) and (OR: 1.39, 95% CI: 0.90–2.15; *P* = 0.14) respectively. These results have been illustrated in Fig. 4.

### Stroke events which were associated with a low versus a high dose of dabigatran

**Fig. 5:**
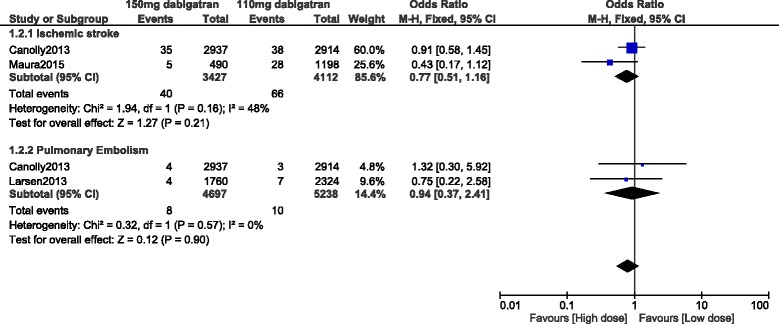
Stroke events associated with 110 mg versus 150 mg dabigatran

Even if ischemic stroke favored a higher dose of dabigatran, (OR: 0.77, 95% CI: 0.51–1.16; *P* = 0.21), the result was not statistically significant. Pulmonary embolism was similarly manifested between these two groups (OR: 0.94, 95% CI: 0.37–2.41; *P* = 0.90) [Fig. 5].

**Fig. 6:**
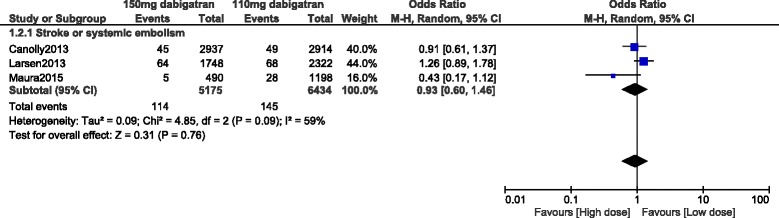
Stoke or systemic embolism associated with 110 mg versus 150 mg dabigatran

Stroke or systemic embolism was also not significantly different between 110 mg and 150 mg dabigatran (OR: 0.93, 95% CI: 0.60–1.46; *P* = 0.76) [Fig. 6].

### Other adverse events

**Fig. 7:**
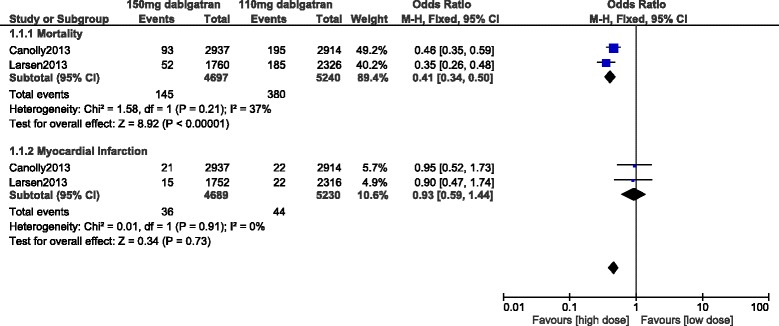
Other adverse outcomes associated with 110 mg versus 150 mg dabigatran

Apart from bleeding and stroke, other important adverse clinical outcomes were also compared. Mortality significantly favored a high dose of dabigatran (OR: 0.41, 95% CI: 0.34–0.50; *P* < 0.00001). However, a similar rate of MI was observed with the use of 110 mg and 150 mg dabigatran, (OR: 0.93, 95% CI: 0.59–1.44; *P* = 0.73) [Fig. 7].

The adverse clinical outcomes which were analyzed have been summarized in Table 4.

**Table 4:** Results of this analysis

Outcomes	OR with 95% CI	*P* value	I^2^ (%)
MI	0.93 [0.59–1.44]	0.73	0
Pulmonary embolism	0.94 [0.37–2.41]	0.90	0
Life-threatening bleeding	1.19 [0.98–1.45]	0.07	0
Extracranial bleeding	1.16 [1.01–1.33]	0.03	0
Fatal bleeding	1.12 [0.69–1.82]	0.65	0
Minor bleeding	1.19 [1.10–1.27]	0.00001	0
Intracranial bleeding	1.24 [0.82–1.88]	0.31	31
Mortality	0.41 [0.34–0.50]	0.00001	37
Intracerebral bleeding	0.85 [0.44–1.65]	0.64	45
Ischemic stroke	0.77 [0.51–1.16]	0.21	48
Major bleeding	1.09 [0.86–1.37]	0.49	57
Stroke or SE	0.93 [0.60–1.46]	0.76	59
GI bleeding	0.93 [0.55–1.58]	0.79	89

Abbreviations: *MI* myocardial infarction, *SE* systemic embolism, *GI* gastro-intestinal

**Fig. 8:**
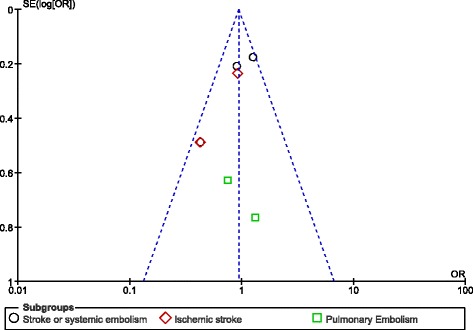
Funnel plots representing publication bias

**Fig. 9:**
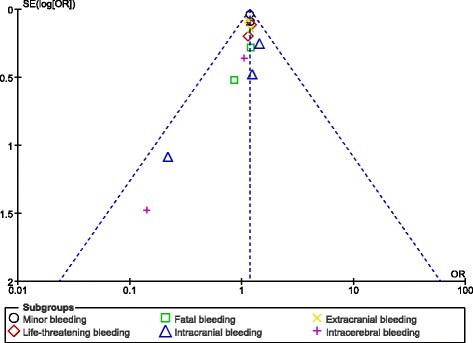
Funnel plots representing publication bias

**Fig. 10:**
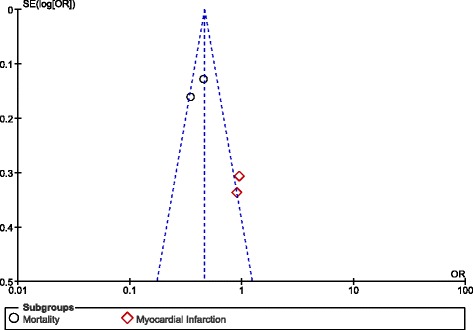
Funnel plots representing publication bias

**Fig. 11:**
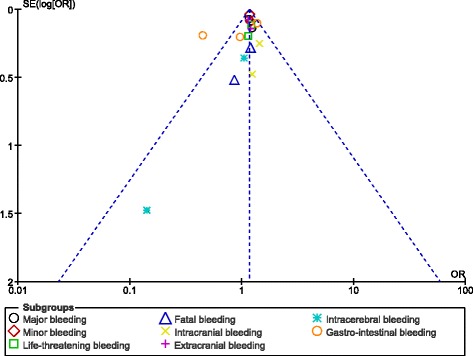
Funnel plots representing publication bias

After visually assessing the funnel plots, a very low publication bias was observed across the studies that assessed several of the adverse clinical events (Figs. 8, 9, 10 and 11).

## Discussion

According to the current practice guideline in United States, dabigatran has been recommended for use as a secondary prevention of stroke in patients who are treated for non-valvular AF. Several studies showed dabigatran to be more effective compared to warfarin, however, the bleeding events associated with 110 and 150 mg dabigatran have seldom been previously studied through meta-analyses.

Dabigatran etexilate is a reversible competitive antagonist of thrombin [10, 11]. Thrombin works by converting fibrinogen to fibrin, cross-linking fibrin monomers via activation of factor XIII and further increasing thrombin production via the activation of factors V and VIII. It also activates platelets in order to initiate several cellular processes.

According to the European (EU) label, a 150 mg dabigatran dosage should be considered the standard or preferred dosage, and should be recommended to all corresponding patients with non-valvular AF, except for those patients who are aged > 80 years, patients who have increased risks of bleeding indicated by a HAS-BLED score of more than 3, and those who are being treated with verapamil at baseline [12]. However, even if a drug to drug interaction has been observed with P-glycoprotein inhibitor verapamil and dabigatran etexilate, whereby the former increased the bioavailability of the latter, this interaction could probably be minimized if verapamil is administered 2-h post dabigatran [13].

Results of this analysis showed that both dosages of dabigatran were associated with similar rates of major and fatal bleeding. However, minor bleeding significantly favored a low dose of dabigatran. Moreover, results for stroke and systemic embolism were not statistically significant. Also, this current result showed a high dose of dabigatran to be associated with a significantly lower mortality rate in these patients who were treated for AF. In addition, when data from observational studies were excluded, 110 mg dabigatran was associated with a significantly lower rate of major and minor bleeding, extracranial bleeding and GI bleeding.

Similar to this analysis, the Randomized Evaluation of Long-Term Anticoagulation Therapy (RE-LY) trial showed a constant rate of several clinical outcomes between these two different dosage regimens of dabigatran. However, a 150 mg dosage was more effective, while a 110 mg dosage was associated with lower major bleeding [14]. Moreover, intracerebral hemorrhage was lower in both groups. Another sub-study from the RE-LY trial showed a higher dose of dabigatran (150 mg) to be associated with significantly fewer strokes while 110 mg dabigatran was associated with a significantly lower rate of major bleeding. But however, these dose regimens were compared to warfarin [15].

In contrast to the current results which showed a higher dosage of dabigatran to significantly reduce mortality compared to a lower dosage, the RE-LY and RE-LY ABLE trials showed a similar mortality rate associated with the different doses of dabigatran [14]. 150 mg dabigatran was associated with a mortality rate of 3.43% and 110 mg dabigatran was associated with 3.55% death per annum. However, it should be noted that in these trials, the follow-up periods were longer, up to 6.7 years, which was not the case in the current analysis. In addition, the fact that 150 mg dabigatran reduced mortality rate by up to 12% whereas 110 mg dabigatran reduced mortality by only 9% when compared to warfarin should also not be ignored [16, 17].

Several other factors should also be considered prior to the use of dabigatran. For example, renal impairment could be a concern in these patients. Research has shown renal impairment to increase the risk of stroke and bleeding in patients with AF. The RELY trial which demonstrated 150 mg dabigatran to be superior compared to warfarin in the prevention of stroke and 110 mg dabigatran to significantly reduce the risk of bleeding among 18,113 patients with non-valvular AF, excluded approximately 80% of patients with renal impairment [18]. It would be worth to know that when these outcomes were investigated in relation to the renal function/impairment, both dosages of dabigatran were consistent. However, when the Cockcroft-Gault, Chronic Kidney Disease Epidemiology Collaboration and Modification of Diet in Renal Disease equations were considered, 110 and 150 mg dabigatran significantly showed a decline in major bleeding rate among patients with glomerular filtration rate greater than 80 mL/min [19]. In addition, the CHADS2 score which is significantly better in predicting ischemic stroke and thromboembolism should also be taken into consideration prior to the selection of an oral anticoagulation therapy for these patients with non-valvular AF [20]. Also, dabigatran use is contraindicated if concomitantly used with other oral anticoagulants, as well as with systemic ketoconazole, cyclosporine, itraconazole, tacrolimus and dronedarone.

This current analysis compared bleeding events and other adverse clinical outcomes which were associated with 110 mg versus 150 mg dabigatran respectively. In terms of anticoagulants, studies showed dabigatran to be an alternative cost-saving drug, compared to warfarin [21]. However, because only a few studies comparing the different dosage of dabigatran in patients with AF have been published, further research is recommended to completely solve this issue.

### Novelty

This analysis is new in several ways. It is among the first meta-analyses comparing bleeding events associated with a low (110 mg) versus a high (150 mg) dose of dabigatran. Since several hospitals still use warfarin, and because research assessing the efficacy and safety of dabigatran is still limited, several interests and concerns would be raised about the application of dabigatran which is available in two different dosages. Will a low dose result in an increased risk of stroke? Or will a high dose result in a high risk of bleeding in patients who are suffering from AF? This analysis is expected to partly provide answers to these questions. However, further research is required to confirm these answers.

### Limitations

Several limitations should be considered in this analysis. First of all, due to a small number of patients which was analyzed, this analysis might not generate robust results. Moreover, an increased level of heterogeneity was observed when analyzing GI bleeding, major bleeding and stroke, and this could be another limitation of this analysis. Several adverse outcomes such as intra-ocular bleeding, pericardial bleeding and so on, were not analyzed since they were reported only in one study. At least two studies were required for comparison. Furthermore, the follow up period was ignored in this analysis, and this might have had an impact on the outcomes and the number of events which occurred, indirectly affecting the results which were obtained.

## Conclusion

No significant differences in major and fatal bleedings were observed with 110 mg versus 150 mg dabigatran. However, 110 mg dabigatran was associated with a significantly lower risk of minor bleeding. These results should further be confirmed in future trials.

## Abbreviations

AF: Atrial fibrillation
FDA: Food and drug administrations
GI: Gastrointestinal
MI: Myocardial infarction
